# Understanding mechanisms of voluntary engagement of mental effort using active inference

**DOI:** 10.3758/s13415-026-01419-z

**Published:** 2026-03-16

**Authors:** Riccardo Maramotti, Thomas Parr, Manuela Tondelli, Daniela Ballotta, Sanjay G. Manohar, Giovanna Zamboni, Giuseppe Pagnoni

**Affiliations:** 1 Department of Biomedical, Metabolic, and Neural Sciences, University of Modena and Reggio Emilia, via Campi 287, Modena, 41125 Italy; 2 Department of Physics, Informatics and Mathematics, University of Modena and Reggio Emilia, via Campi 213/B, Modena, 41125 Italy; 3 Department of Mathematics and Computer Sciences, University of Ferrara, via Machiavelli 30, Ferrara, 44121 Italy; 4 Nuffield Department of Clinical Neurosciences, University of Oxford, Oxford, OX3 9DU United Kingdom; 5 Neurology Unit, Baggiovara Hospital, via Giardini 1355, Modena, 41126 Italy

**Keywords:** Mental effort, Active inference, Stroop task, Motivation, Cognitive control, Computational model

## Abstract

The voluntary investment of mental effort is an understudied aspect of cognitive control, whose underlying mechanisms remain poorly understood. Here, we investigated this process using a computational model of the Stroop task within the framework of active inference. In the Stroop task, participants must report the font color of a presented color name, while suppressing the automatic tendency to read the word itself. In this study, we asked twenty healthy young adults to perform the Stroop task under two conditions: with maximum exertion or as relaxed as possible. Their behavior was modeled using a two-layer generative model grounded in active inference, conceptualizing cognitive effort as the extent to which habitual response tendencies are overridden by goal-directed behavior. This approach enabled the estimation of two key latent parameters: (i) the individual’s habitual bias toward word reading over color naming and (ii) the degree of motivation to perform the task correctly. Our findings indicate that voluntary engagement of maximal effort was associated with an increased preference for correct performance, whereas its relationship with the habitual bias toward word reading did not show a consistent group effect. These results support the hypothesis that the voluntary investment of cognitive effort is primarily governed by an increased motivation for accuracy rather than by the direct inhibition of habitual response tendencies. This computational approach holds potential relevance for clinical settings where impaired intentional effort allocation is observed in psychiatric and neurological disorders.

**Supplementary Information:**

The online version contains supplementary material available at 10.3758/s13415-026-01419-z.

## Introduction

Mental effort is a widely used concept in daily life, often associated with willpower and self-control. Students are told to put more effort into doing their homework. Adults may have to learn the appropriate balance in their job between insufficient engagement and excessive exertion that may result in burnout. Despite its common use, mental effort remains a somewhat elusive concept in cognitive neuroscience (Shepherd, 2023). Part of the problem is that mental or cognitive effort (we will use these terms interchangeably here) has at least three distinct connotations (Bruya and Tang, 2018; Khachouf et al., 2017; Shepherd, 2023; Wolpe et al., 2024). First, it may refer to how many cognitive resources are automatically recruited by a task, which varies according to task difficulty. This aspect of effort – which, for convenience, we will call *exogenous* effort and which is not necessarily conscious – was the subject of Daniel Kahneman’s seminal work (Kahneman, 1973), where it was essentially equated to attentional allocation (see also Sarter et al., 2006). The second aspect of effort, which we will call *endogenous*, is an executive one, related to self-control and the ability to voluntarily modulate the degree of engagement in a demanding task (Muraven and Baumeister, 2000; Shepherd, 2023). The third dimension of effort is an *affective*, consciously experienced one (Robinson and Morsella, 2014), usually related to the aversive feeling (but not always, see Inzlicht et al., 2018; Székely and Michael, 2021; Carruthers and Williams, 2022) associated with the performance of a laborious undertaking (Kurzban, 2016; Morgan, 1994; Székely and Michael, 2021). These three aspects may not be easily dissociable and are often closely linked, as exemplified by the finding that emotional arousal not only facilitates physical effort but also decreases the perception of effort (Schmidt et al., 2009).

Two other factors are deeply woven into the fabric of mental effort: individual motivation and the foreseen consequences of our actions. Several researchers have argued for an intrinsic relationship between the amount of cognitive effort invested in a task and its expected reward, within a cost-benefit computational framework (Bénon et al., 2024; Croxson et al., 2009; Manohar et al., 2015; Shenhav et al., 2017), often from a neuroeconomic perspective (Kool and Botvinick, 2018). A key role appears to be played by dopaminergic transmission and mesolimbic circuits (Salamone et al., 2016; Walton et al., 2003; Walton and Bouret, 2019; Walton et al., 2006; Westbrook and Braver, 2016; Westbrook et al., 2020), although cholinergic, noradrenergic, and serotonergic processes are also likely involved (Hosking et al., 2015). Other scholars have proposed that the subjective feeling of effort results from a prediction of the degree to which the ongoing task will disrupt homeostasis (Noakes, 2012; but see Inzlicht and Marcora, 2016).

The active inference approach licenses a fresh outlook on the topic of cognitive effort, by defining it as a divergence between the probability distribution over the courses of action (*policies*), conditioned on the current context, and the probability distribution over the same policies that reflects our habitual behavior (Parr et al., 2023). Put more simply, whenever we perform a demanding task, we face a tension between the behavioral strategy we would follow ‘automatically’, and the one that is required for the correct performance. This tension, or divergence, is taken as a direct measure of the amount of effort associated with the performance. This approach is in line with descriptions of decision-making as a process that pits effortful policies against habitual ones (Dickinson, 1985; Kahneman, 2003), which have been more recently cast in terms of model-based vs. model-free strategy selection (see Kool et al., 2017, 9). While this proposed definition is based upon previous literature, there is a wide range of definitions of effort, and we acknowledge this will not be compatible with all of them. It is perhaps more accurate to say that this is an operationalization of the notion of effort.

Among the many experimental psychology paradigms designed to contrast habitual responses, one of the most widely used is the Stroop color–word interference task. In this task, participants are shown words that represent color names printed in different colors. In the ‘word’ condition, they are asked to read (or signal via an appropriately coded response device) the words, that is, to report the displayed text. In the ‘color’ condition, participants are asked to report the font color of the presented word. Crucially, in different trials, the text and font color may be *congruent* (i.e., the word ‘RED’ in red fonts), or *incongruent* (i.e., the word ‘RED’ in green fonts). Given our habitual tendency to read words, responding to word stimuli by stating their font color is a policy that requires more effort (in all of the three meanings described above) compared to the natural action of reading the word text.

An active inference model of the Stroop task was recently shown to reproduce various characteristics of actual behavioral and neurophysiological data (Parr et al., 2023). The model relied on two key parameters, *c* and *e*, which were used to represent the participant’s motivation to perform the task correctly and the strength of their habitual tendency to read words (rather than name their colors), respectively. In the present study, we used empirical behavioral data, collected from a sample of volunteers performing the Stroop task, to invert an adapted version of that model and recover individual estimates of the *c* and *e* parameters. Crucially, we introduced a modification of the experimental paradigm: participants were asked to perform the task on different blocks either “with maximum exertion” or “as relaxed as possible”. This modification follows a previous neuroimaging study (Khachouf et al., 2017) that demonstrated measurable differences in both behavior and functional imaging findings with this intervention. In addition to the *exogenous* effort demands of the classical Stroop task, we thus introduced an *endogenous* effort component as an explicit experimental manipulation by instructing participants to apply varying levels of voluntary effort.

**Fig. 1:**
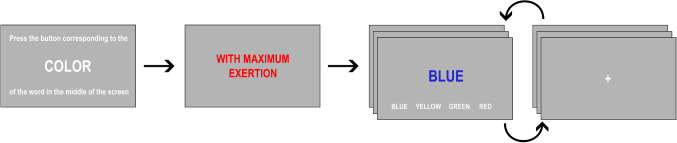
Structure of an experimental block. Prior to each block, participants were informed by textual cues about the target (“respond to the text” or “respond to the font color”) and the degree of effort they were expected to invest in it. The word stimuli were presented centrally, with a bottom row of color name labels (*in white ink*) reminding the participant of the position of the corresponding buttons on the response box

The aim of the present study was to investigate the processes underlying people’s responses to encouragement to exert more effort. More specifically, we sought to assess the relative evidence for two hypotheses about the processes underwriting the intentional engagement of cognitive effort: is voluntary effort mediated by (1) increasing the motivation for an optimal performance (e.g., “I’ll do the task as if it was the most important thing today”), or (2) suppressing the automatic habitual response (i.e., reading the word)? A further two hypotheses that follow from this are that both (1) and (2) may be in play, or that neither (1) nor (2) provide adequate explanations for the deployment of voluntary effort. The analysis we applied consists of a standard Bayesian inference approach to fit a small number of psychologically interpretable parameters using a previously published model of the Stroop task. This parameter estimation was followed by hypothesis testing using Bayesian model selection to compare the four hypotheses represented by reduced versions of our full model. Such approaches are common in neuroimaging analyses – and in fact use the same software routines as Dynamic Causal Modeling. In the Methods section, we set out the key details and intuitions that the reader will need to understand the results of our analysis.

While this study is behavioral, with no direct neural measurements, and limited in terms of what we can say about the neural underpinnings of effort, there is a wide literature relating concepts of effort to specific aspects of brain anatomy and physiology, to which we will relate in the Discussion section. Of particular interest to us is the Khachouf et al. (2017) fMRI analysis, which employed the task setup that served as the basis for our paradigm.

## Methods

### Participants

Twenty volunteers (12 females; mean age: 27.9 ± 5.7 years; range: 18–43 years) took part in the study. A history of psychiatric or neurological disorders and current use of psychoactive medications were considered exclusion criteria. The study was carried out according to the 2013 version of the Declaration of Helsinki, after approval by the local Ethics Committee (protocol number: CEAR 2024/0144289). Written informed consent to participate in the study was obtained from all volunteers.

### Experimental design

We employed a version of the color–word Stroop task, using a finger-press response modality via a button box. Participants were instructed to focus on visual stimuli presented on a laptop screen using the PsychoPy software (Peirce et al., 2019). The stimuli consisted of four colored words (‘RED’, ‘GREEN’, ‘YELLOW’, and ‘BLUE’), which were displayed either in a semantically matching font color for *congruent* trials (e.g., the word ‘RED’ in red fonts), or in a non-matching font color for *incongruent* trials (e.g., the word ‘RED’ in green fonts). The Stroop interference effect, which is deemed to reflect effortful cognitive control, refers to participants exhibiting longer response times and a higher number of errors during incongruent trials, compared to congruent ones.

Each participant completed four runs of the experimental task. Each run consisted of 96 trials, yielding a total of 384 trials. The intertrial interval (between a response and the onset of the following stimulus) was set at 1 s, and the participants were not constrained by a time limit for responding. Each run was divided into four blocks of 24 stimuli. In two of these blocks, participants were asked to respond to font color, while in the other two, they were instructed to respond to the written text (Fig. 1).

Crucially, as in Khachouf et al. (2017), the participants were instructed to perform alternating runs with two distinct levels of effort: (a) “with maximum exertion” (EXERT condition) or (b) “as relaxed as possible” (RELAX condition). As a consequence, our experimental design had 3 factors: *effort* (EXERT or RELAX), *target* (word or color) and *congruency*. Note that the instructions to the participants focused on differentiating their mental attitude adopted in performing the task, rather than on achieving a better (more accurate and fast) performance in the EXERT vs. the RELAX condition. Thus, the ensuing changes in behavioral responses can be attributed to the participants’ attempt to execute an intentional modulation of effort. The instructions were displayed in the center of the screen for a period of 2 seconds at the beginning of each block, reminding the participants to put in high or low effort right before performing the task (Fig. 1). To avoid order effects, runs and blocks were counterbalanced across participants. In order to ensure full comprehension of the task, all participants completed a practice run prior to the start of the actual data collection.

### Subjective task-load ratings

At the end of each run, participants were asked to rate their subjective workload using the NASA-TLX rating instrument (Hart and Staveland, 1988). This consists of six questions, presented in random order, addressing the following phenomenological dimensions:

1. *Mental demand*: how much thinking, deciding, or calculating was required
2. *Physical demand*: the amount and intensity of physical activity required to complete the task
3. *Temporal demand*: the amount of time pressure involved in completing the task
4. *Effort*: the degree of exertion required to maintain the participant’s performance level
5. *Performance*: the perceived level of success in completing the task
6. *Frustration level*: how insecure, discouraged, or content the participant felt during the task

Each question was displayed in the center of the screen, followed by a horizontal line labeled ‘very low’ on the left and ‘very high’ on the right. Participants responded by positioning a cursor on the line and their selection was subsequently converted to a score of 0 to 10 (inverted scores were used for the *Performance* reports).

### Descriptive and basic statistics of collected data

Accuracy and response time data were examined via standard descriptive statistics, stratified by all experimental conditions. To confirm the presence of the Stroop interference effect (*congruent* vs. *incongruent*), we performed a three-way repeated measures ANOVA on response times.

To verify the effectiveness of the experimental manipulation, we performed paired *t* tests comparing the average NASA-TLX ratings of the EXERT and RELAX blocks. This was done separately for each of the six NASA-TLX dimensions, and the results were corrected for multiple comparisons using the Bonferroni method, with a significance threshold set at $p_{corr} < 0.05$.

### Active inference model of the experimental task

We adopted a modeling approach based on the theoretical framework of active inference (Parr et al., 2022). Active inference is based on the idea that our brains make use of internal generative models to predict sensory data and guide behavior. By ‘inverting’ these models, our brains draw perceptual inferences about the causes of observed data and generate behavior that ensures future data comply with prior beliefs. The key thing to know about this framework is that behavior depends upon the form and parameters of the internal model assumed to be used by the brain (i.e., we are modeling how the brain models the world; Daunizeau et al., 2010).

The model we implemented replicates the structure of the Stroop task itself, dealing with two timescales (that of the response to an individual stimulus and the response to a stream of stimuli under a given instruction) that explicitly match the modeling to the experimental design. Our model is based on a recently proposed active inference framework for the Stroop task (Parr et al., 2023), with important adaptations tailored to our experimental design. The detailed structure of the model is presented in Supplementary Materials, replicating the key figures of Parr et al. (2023), and walking readers through the technical aspects of this model. This also includes a posterior predictive check and a parameter recovery analysis demonstrating the validity of our modeling approach.

In active inference, the potential actions of participants in response to task instructions are represented by alternative *policies* $\boldsymbol{\pi }$.^1^ These policies can be viewed as probabilistic beliefs about the type of response to the issue, which subsequently determine the button pressed on the button box. In our case, there are just two available policies, i.e., “report the word text” or “report the font color”. Habitual actions – word reading, in this case – are represented by assigning a higher prior probability, which translates to being ‘easier’ to perform. In contrast, non-habitual actions – like font-color naming, here – are encoded with a lower prior probability, which corresponds to the requirement of a greater cognitive effort.

Our analysis focused primarily on two parameters, namely *c* and *e*, which reflect the motivation to perform the task well and the habitual bias towards reading the word (*vs*. stating the font color), respectively. Higher values of *c* indicate a stronger preference for accurate performance, while higher values of *e* indicate a greater strength of the habit to automatically read the word (and thus the need for increased cognitive effort to suppress this habitual response). The interaction between these parameters reflects various individual scenarios, such as cases where a strong motivation for accuracy (*c*) can mitigate the impact of a strong habitual tendency (*e*) toward word reading over font color naming.

#### Response choice

The generative model enables simulation of response choices as *actions*. Instead of simply selecting the most probable action, actions are generated by sampling from a probability distribution $\textbf{u}$, given by the expected observation at the next time step (itself determined by averaging observations conditioned upon policies under a distribution $\boldsymbol{\pi }$ that scores alternative policies based upon their expected free energy for the next time step). A softmax function^2^$\sigma$ is applied to the log-distribution of the observations, that is also weighted by a parameter $\lambda$ to account for uncertainty in action not captured purely by this observation distribution:

1 $$\begin{aligned} \textbf{u}_{t+1} :\,\,=\,\, \textbf{u}(c, e, \lambda ) = \sigma \left( \lambda \ln \sum _\pi \boldsymbol{\pi }_\pi \cdot \boldsymbol{o}_{\pi ,t+1} \right) \qquad \lambda \in \mathbb {R}^{+} \end{aligned}$$

Effectively, this means that the next controllable observation $o_{t+1}$ – i.e., the button press – is sampled from $\boldsymbol{u}_{t+1}$. The parameter $\lambda$, typically referred to as *inverse temperature*, regulates the level of stochasticity in action selection. Higher values of $\lambda$ result in more deterministic actions, whereas lower values increase variability in decision-making.

#### Response times

Response times can also be simulated by the model, based on the agent’s confidence in her response choice, following a common approach in drift-diffusion models of decision-making (Ratcliff and McKoon, 2008). More specifically, the response time is modeled here as a function of the entropy of the predicted response choice distribution at the next time step, $H_{t+1} = -\textbf{u}_{t+1} \cdot \ln (\textbf{u}_{t+1})$. Higher entropy values correspond to longer response times:

2 $$\begin{aligned} &  r_t :\,\,=\,\, r(c, e, \lambda , \alpha ) = \exp (\alpha ) \exp (n + H_{t+1}) \nonumber \\ &  n \sim \mathcal {N}(0, \frac{1}{16}) \qquad \alpha \in \mathbb {R} \end{aligned}$$

In this equation, the entropy term can be interpreted as the logarithmic drift rate that governs decision time, with the Gaussian random variable *n* accounting for the stochastic component of diffusion (Ratcliff and McKoon, 2008). The constant $\exp (\alpha )$ represents the baseline response time under conditions of maximum confidence, where entropy approaches zero. In essence, $\alpha$ represents the minimum expected response time, i.e., when the subject is fully certain about her response choice; at each trial, within the active inference model, this minimum reaction time is adjusted by an amount represented by the entropy *H*.

#### Simulated behavior

A characteristic feature of many behavioral tasks, including the Stroop, is a speed–accuracy trade-off, whereby attempting to respond more quickly often causes a decrease in accuracy. Since the question of whether investing more effort in a task affects speed, accuracy, or both is a relevant one, it is important that the simulated behavioral data from our generative model exhibit a realistic relationship between speed and accuracy.

**Fig. 2:**
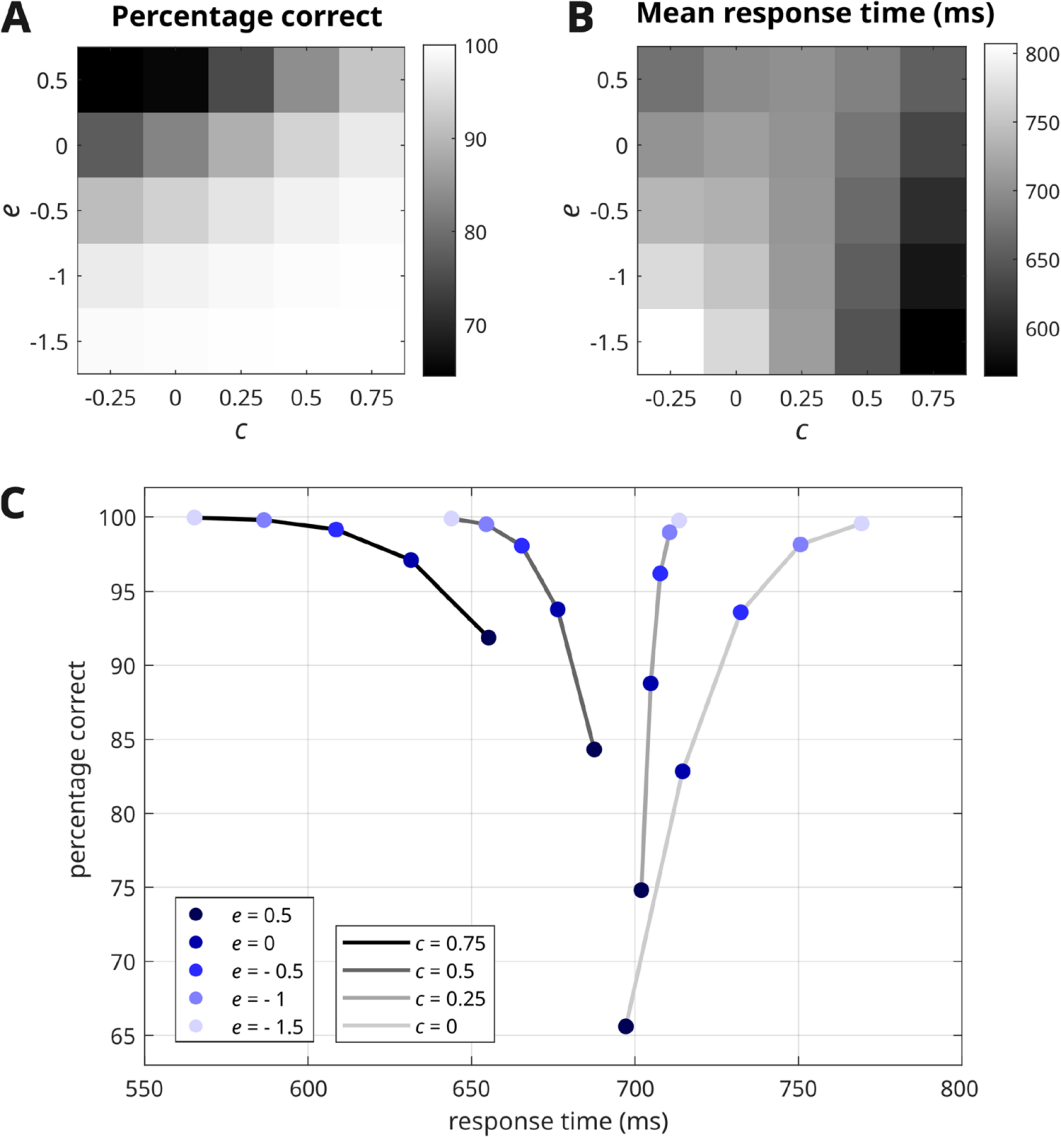
Panels **A** and **B** illustrates the average accuracy and response times for simulated data under different prior beliefs. Panel **C** integrates this information into a two-dimensional plot, illustrating the relationship between speed and accuracy across different fixed levels of motivation (*c*) and habit (*e*). The gradient of the curve may be either positive or negative, indicating that an increase in speed can be associated with either a decrease or an increase in accuracy. In all these simulations, the other parameters were set as follows: $\lambda = \frac{1}{4}$, $\alpha = \ln (\frac{1}{2})$, which correspond to the values used in the simulations from Parr et al. (2023). Note that the graph includes some zones of very poor accuracy, which correspond to high values of *e* and low values of *c*. As our actual behavioral data did not exhibit such low levels of performance, we did not expect to recover the corresponding combination of parameter values, and indeed, these were not obtained (see Results)

Figure 2 shows the accuracy and response times of the data generated by the model using various values of the parameters *c* and *e*. In panels A and B, when *c* is slightly below 0 (leftmost columns), an increase in *e* results in decreased accuracy and faster response times. This means that when the motivation for performing the task well is low, the presence of strong habitual behaviors that are discordant with the task requirements will prevail, producing quick but inaccurate responses. In contrast, in the rightmost columns of the grid plots, where *c* is greater than 0, increasing *e* values lead to a (small) decline in both accuracy and response speed. In other words, when motivation is strong, we will generally observe fast and accurate responses, with only slight decreases in performance as the cognitive demands of the task (in terms of its deviation from habitual behavior) increase. Panel C of Fig. 2 illustrates more explicitly the speed–accuracy trade-off for various values of the *c* and *e* parameters.

### Model fitting and parameter estimation

We now turn to the central aim of the present study: to infer the values of hidden causal factors of behavior from measures of task performance and use these to test hypotheses. In our case, this involves estimating the values of parameters *c*, *e*, $\lambda$, and $\alpha$ for each participant, by fitting the generative model to the observed data (response choices and response times) via a variational Laplace procedure (Zeidman et al., 2023).

#### Single-subject level

Our modeling approach uses as data not only the overall accuracy of the responses, but also the sequence of choices (i.e., button presses), allowing for sequential effects to inform model fitting. Therefore, the log-likelihood $\mathcal {L}$ we used for model inversion depends on both response choices and response times, according to the following formula:

3 $$\begin{aligned} \mathcal {L}(r_t, o_t,c,e,\lambda , \alpha )= &  \underbrace{\sum _t \ln (o_{t} \cdot \textbf{u}_{t-1})}_{\text {choices}}\nonumber \\ &  - \underbrace{\sum _t \frac{1}{16} (\ln r_t + \textbf{u}_t \cdot \ln {\textbf{u}_t} - \alpha )^2}_{\text {response times}} \end{aligned}$$

where $o_{t}$ denotes the observed response choice (as a one-hot vector^3^) and $r_t$ denotes the response time at time step *t*.

Bayesian inference requires the definition of prior distributions for the model parameters. In our case, all prior distributions were chosen as Gaussian, resulting in normal posteriors. The prior means of *c* and *e* were set at 0. In this way, the prior preference for being correct was approximately 7.4 times the prior preference for being incorrect, while 65% of the time the participant was expected to read the word (and only 35% of the time to name the color). In order to ensure the positivity of the parameter representing the stochasticity in the model, we defined $\lambda = \exp (\zeta )$ and modeled $\zeta$ using a prior mean of $\ln (\frac{1}{4})$. Finally, the parameter $\alpha$ (associated with the lower bound of response times) was modeled using a prior mean of $\ln (\frac{1}{2})$. Regarding the variance of these Gaussian priors, we used $\frac{1}{4}$ for all parameters. For a detailed explanation of these prior choices, please refer to Supplementary Materials. Data from the EXERT and RELAX runs were used to fit the model separately for each of the 20 participants, yielding a total of 40 sets of model parameters.

#### Group-level

The group-level analysis aimed to determine whether the motivation parameter (*c*) and the bias parameter (*e*) differed between the two effort conditions. To address this, we applied parametric empirical Bayes (PEB; Friston et al., 2016), which updates individual estimates and predicts group-level *c* and *e* parameters, representing the difference between the EXERT and RELAX conditions. This approach mitigates overfitting and is well suited for small sample sizes.

**Fig. 3:**
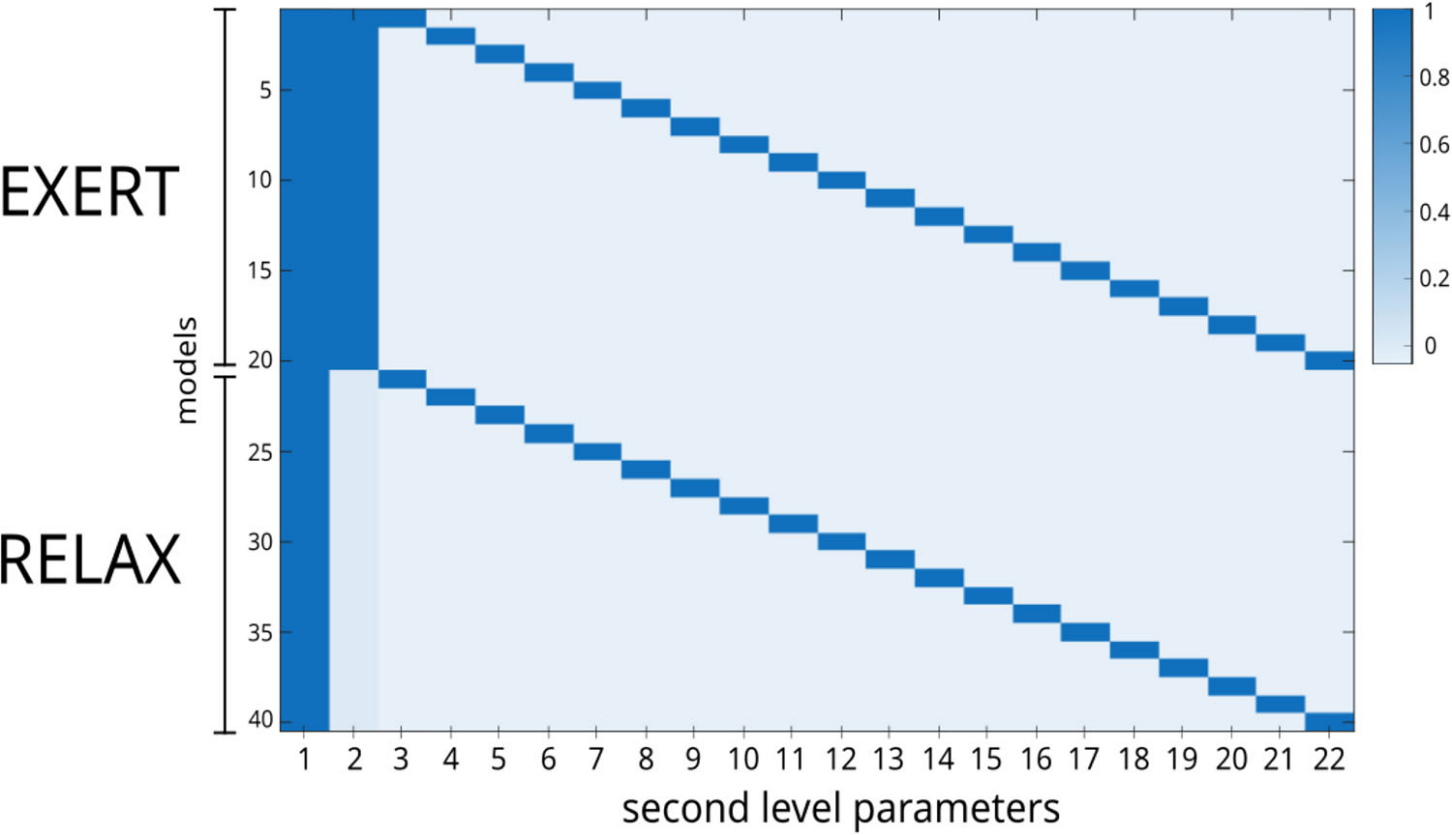
Design matrix of the PEB analysis. The *first column* represents the intercept, while the *second column* refers to the effect of interest, which is the effect of intentional effort on motivation *c* and bias *e*. The first 20 rows represent the parameters relative to the EXERT data, and the last twenty rows the parameters relative to the RELAX data

PEB requires the specification of a design matrix to account for sources of variability between subjects. While the first column contains all ones and represents the intercept, the second column typically refers to the effect of interest; in our case, it contained 1 for EXERT models and 0 for RELAX models. The remaining columns captured the subject-specific variability and were mean-centered (Fig. 3).^4^

Bayesian Model Reduction (BMR; Friston et al., 2016) tests reduced versions of the full model obtained with PEB, selecting the most plausible one and highlighting key group-level effects. We used BMR to compare four models: the *full model*, which includes the effect of voluntary effort on both *c* and *e*; a *null model* with no effect of voluntary effort on either parameter; and two models with the effect of voluntary effort on only one parameter. In the BMR scheme, if a model has a posterior probability greater than 90% its parameter estimates are taken as the final values. Otherwise, the final estimates are computed as a weighted average of the estimates from all the models, where the weights are the posterior probabilities of the models (i.e., Bayesian model averaging). Credible intervals are also computed using this procedure, thus we considered a posterior probability of being non-zero higher than 90% as a criterion for reasonable evidence of the effect of voluntary effort on the specified parameter. This allowed us to test if voluntary effort is mediated by (1) increasing motivation for being correct, (2) suppressing the automatic habitual response, by both (1) and (2), or by neither (1) nor (2).

**Table 1:** Average values for the observed accuracy and response times (RT) across all eight conditions. For response times, within-subjects 95% confidence intervals (CI) are also shown

Effort	Target	Congruency	Average accuracy	Average RT	RT 95% CI
EXERT	color	congruent	98.2%	592 ms	535 - 653 ms
EXERT	color	incongruent	95.7%	717 ms	652 -787 ms
EXERT	word	congruent	98.4%	609 ms	559 - 656 ms
EXERT	word	incongruent	96.8%	706 ms	642 - 766 ms
RELAX	color	congruent	98.0%	694 ms	632 - 756 ms
RELAX	color	incongruent	94.0%	859 ms	779 -939 ms
RELAX	word	congruent	98.0%	699 ms	641 - 758 ms
RELAX	word	incongruent	96.7%	806 ms	723 - 880 ms

## Results

### Descriptive and basic statistics of collected data

#### Accuracy and response times

**Fig. 4:**
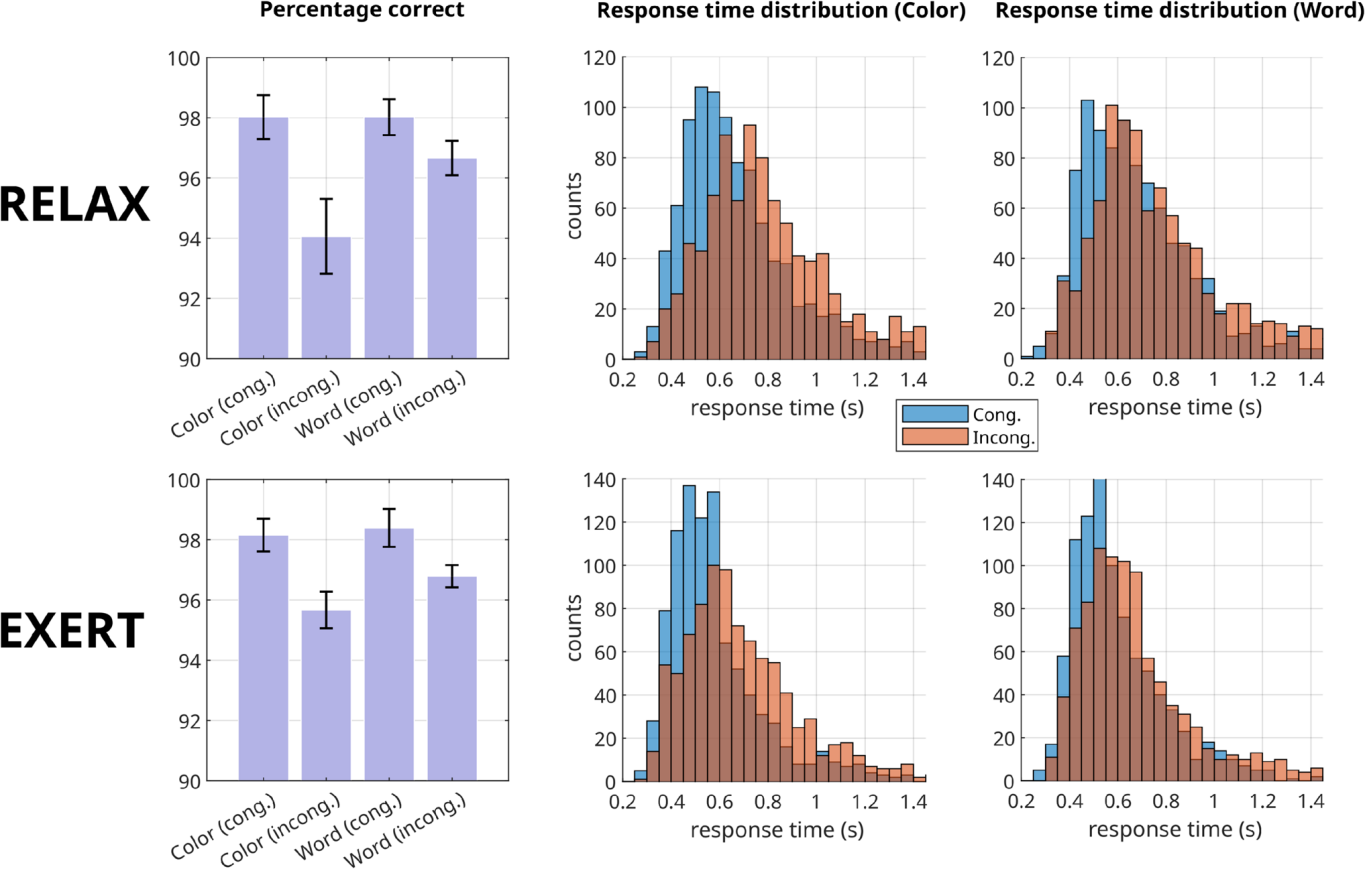
Graphical representation of the observed behavioral data. The left column illustrates accuracy across the various conditions (*error bars* represent within-subject standard errors), while the remaining two columns display the distributions of the observed response times for the two tasks (‘report the font color’ and ‘report the word text’). For the results of a repeated-measure ANOVA on response times, see Supplementary Materials

The observed accuracy and response times are presented in Table 1 and Fig. 4. In both the EXERT and RELAX blocks, the *color incongruent* condition – where participants had to respond to the font color and the stimulus was incongruent – yielded the lowest accuracy and the longest response times. Performance in the *word incongruent* condition – where participants had to respond to the stimulus text and the stimulus was incongruent – was comparatively better, even if it remained slower and less accurate than in all *congruent* conditions. In the RELAX condition, response times were generally slower compared to the EXERT condition, although a relevant reduction in accuracy was only observed in the color-incongruent condition (94.0% vs. 95.7%). Results of a three-way repeated measures ANOVA performed to confirm the Stroop interference effect on response times are reported in the Supplementary Materials.

#### Subjective task-load measurements

Three of the six NASA-TLX dimensions had statistically significant differences between the EXERT and RELAX blocks. Specifically, higher values for the EXERT, compared to the RELAX, runs were reported for *mental demand* (mean = 6.57 vs. 5.15, $\Delta = 1.42$, 95% CI [0.82, 2.03], $p < 0.001$), *temporal demand* (mean = 5.24 vs. 3.87, $\Delta = 1.37$, 95% CI [0.75, 2.00], $p = 0.001$), and *effort* (mean = 6.74 vs. 4.83, $\Delta = 1.91$, 95% CI [1.29, 2.52], $p < 0.001$).

### Model fitting and parameter estimation

**Fig. 5:**
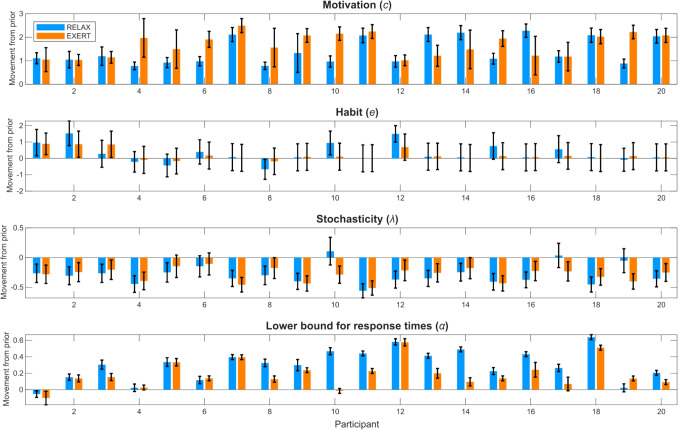
Posterior estimates and credible intervals for the parameters *c*, *e*, $\lambda$, and $\alpha$ in terms of their deviation from prior values

Figure 5 illustrates the model’s parameter estimates across subjects. We did not observe any significant correlation between the NASA-TLX ratings and the estimated values for the *c* and *e* parameters – both separately for the EXERT and RELAX conditions, and for the EXERT-RELAX differences (see Supplementary Materials).

**Fig. 6:**
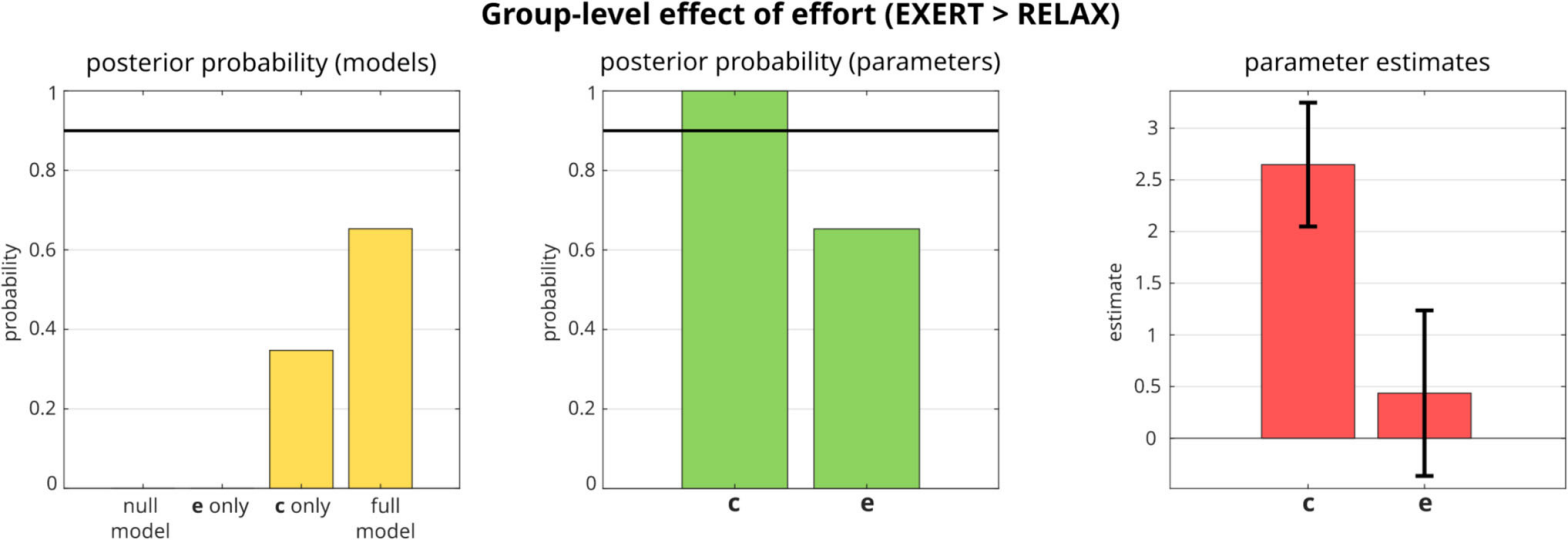
Results of group-level analysis. BMR was used to compute posterior probabilities of four models: a ’full model’ that included both *c* and *e*, a ’null model’ that excluded both, and two models that included only one of the parameters. The *left panel* shows the posterior probability for each model, with a *horizontal line* indicating the 90% probability level. The *middle panel* displays the posterior probability of being non-zero for each second-level parameter (representing the difference in *c* and *e* between EXERT and RELAX blocks). These values are computed as the sum of the posterior probabilities of the models where these parameters are present; for example the posterior probability of group-level *c* is the sum of the posterior probabilities of the full model and the ’only *c*’ model. Note that the full model has a larger posterior probability than the one that includes only the *c* parameter, providing some evidence for the relevance of both parameters in explaining the data. However, this can be nuanced by computing final parameter estimates from a weighted average (i.e., Bayesian model averaging), where the weights are the posterior probabilities of the models. The *right panel* shows these weighted averages with 90% credible intervals. From this graph, we can see that the final posterior probability distribution for group-level *e* parameter has 90% credible intervals that include zero. As such, if we apply an arbitrary 90% thresholding, we would be unable to conclude that this parameter differs between EXERT and RELAX condition

For the group-level analysis, we employed parametric empirical Bayes (PEB) followed by Bayesian model reduction (BMR), to investigate how the *c* and *e* parameters can explain the effect of intentional investment of effort in the task. This analysis identifies the model that best explains the observed differences between the EXERT and RELAX conditions. The left panel of Fig. 6 shows that, although the ’full model’ that includes the effect of effort on both the *c* and *e* parameters exhibits the highest posterior probability (65.3%), the model with effort affecting only the *c* parameter also demonstrates a substantial posterior probability (34.7%), while the model considering only *e* has a negligible posterior probability. As all models have a posterior probability less than 90%, the final parameter estimates are computed as a weighted average of the estimates from models, where the weights are their posterior probabilities.

Therefore, *c* is the only parameter whose variation with respect to endogenous effort has a probability of being non-zero higher than 90% (Fig. 6, middle and right). This finding suggests that the intentional engagement of effort primarily affects motivation, as the parameter *c*, in contrast to the parameter *e*, is significantly higher in the EXERT condition compared to the RELAX one. Nevertheless, the findings of this group-level analysis do not preclude the possibility that, for certain individuals, endogenous effort may be mediated by changes in the *e* parameter.

## Discussion

We studied the effects of intentional investment of mental effort, using a Stroop task and asking participants to perform it with maximum exertion (EXERT) or as relaxed as possible (RELAX), in alternating runs. Incongruent stimuli in the classical Stroop task already require a degree of cognitive effort to suppress the automatic tendency to read the text in favor of identifying the font color. Thus, in addition to this *exogenous* effort, which reflects the cognitive load imposed by task demands, our experimental design introduced an explicit *endogenous* component of effort, namely a voluntary modulation of the participant’s investment in performing the task. While exogenous effort is driven by task difficulty, endogenous effort involves self-regulation and intentional control. As the experimental conditions EXERT and RELAX differed only in the instruction about *how* to do the task (not about *what* to do), the observed behavioral differences between the two conditions can be interpreted as reflecting the cognitive processes associated with the attempt to intentionally vary the degree of invested mental effort.

Parr et al. (2023) developed an active inference model of the word–color Stroop task simulating known features of task performance under a novel conceptualization of the construct of mental effort. We used a slightly modified version of this model and fitted it to actual performance data from the Stroop task. This approach allowed us to evaluate the relative evidence of two hypothesized mechanisms of intentional effort: a weakening of the compulsive power of habitual policies, on the one hand, and an increase of intrinsic motivation, on the other. One could ask why, given that many Stroop paradigms make use of only the color-naming condition, we have elected to also include a word-reading condition. The reason for this is that, in estimating the *e* parameter, dealing with the habitual effect of word reading, it is useful to be able to vary the demands placed on this parameter over and above the effects of some trials being incongruent.

The results showed that voluntary engagement of effort in the EXERT condition was associated with a significant increase in the motivation parameter (*c*) only, suggesting that what participants do when asked to engage maximum effort is to endogenously intensify their motivation, possibly by modulating precision weighting of connections in reward circuits. This seems plausible as the alternative – i.e., modifying directly the strength of our behavioral habits – may not be feasible in the sense that we may not have direct, operational access to the relevant mechanisms (or more simply that modifying habit strengths requires a longer time frame and cannot be performed in real time). It is also possible that this autonomous intensification of motivation is implemented by activating reward-related processes (not necessarily in an explicit, conscious manner), which would align with experimental data showing that increasing the magnitude of a reward leads to greater effort investments (Camerer and Hogarth, 1999; Jimura et al., 2010) and improves executive function (Krebs et al., 2010).

It is important to qualify some of the language used. Statements about motivation and demand here refer explicitly to the inferred parameters *c* and *e*, which may or may not reflect commonly held psychological definitions of these attributes – although we suggest that they do reflect a formalization of at least some definitions. In other words, the statement that an instruction to voluntarily exert oneself led to an increase in their motivation is really shorthand for saying that it led to an increase in the estimate of the *c* parameter that best explained their behavior. The meaning of the *c* and *e* parameters comes from their influence over decision-making in the models in which they appear. The former determines the degree to which a decision is made to maximize the probability of a particular outcome, while the latter determines the degree to which a decision is biased in a context-independent manner, which may have been accumulated following repeated performance of those same decisions over time. Recent research on cognitive control has shown that different control processes do not necessarily exclude each other and may act in parallel (Gheza and Kool, 2025; Ritz and Shenhav, 2024). Indeed, our model does not posit, by design, a trade-off between motivation and habit; the corresponding parameters were implemented as distinct causal factors. As in any model of this kind, however, when fitting the model to empirical data, a certain degree of dependency among parameters may arise—meaning that changes in the fitted value of one parameter may be accompanied by adjustments in others. Outside of the Stroop task, similar parameters have been estimated in the context of motivated decision-making tasks, including in study of substance abuse disorders (Hakimi et al., 2024), pharmacological studies of serotonergic function (Fisher et al., 2024), and even in saccadic exploration tasks (Mirza et al., 2018).

Although the mechanisms corresponding to the hypotheses cited above can both be seen as instances of *mental action* – i.e., precision modulation from the point of view of active inference (Limanowski and Friston, 2018; Sandved-Smith et al., 2021) – they differ arguably in the neural locations where precision changes would be respectively implemented. In a previous fMRI study with a similar experimental design, Khachouf et al. (2017) observed significant activity changes triggered by the instructional cue to apply intentional effort to the Stroop task in a wide mosaic of brain regions, including areas belonging to the salience network (anterior/middle cingulate and anterior insula cortex), to the fronto-parietal attentional network (superior parietal cortex, supplementary and pre-supplementary motor area, frontal eye fields and superior frontal gyrus, dorsolateral prefrontal cortex), to the corpus striatum of the basal ganglia, and to the midbrain arousal system. Research on the neural bases of motivational processes has consistently implicated the circuits supporting salience detection, attentional control and reward (Di Domenico and Ryan, 2017; Parro et al., 2018), with a particular focus on dopaminergic transmission (Salamone and Correa, 2024; Treadway and Salamone, 2022). In the clinic, the emergence of apathy in neurological conditions – especially in Parkinson’s and Alzheimer’s diseases, but also in stroke – has been associated with functional impairment and anatomical atrophy in many of the same regions, in particular the medial frontal cortex and the striatum (Le Heron et al., 2018; Levy and Dubois, 2006). Parr et al. (2023) proposed a tentative mapping of the model’s architecture onto a subset of the brain regions listed above – see Fig. 4 in the cited reference. Also, a recent study using a transcranial stimulation protocol during an N-back task, demonstrated the causal role of dorsolateral prefrontal cortex in motivating the engagement of effortful cognitive control (Soutschek and Tobler, 2020).

On this basis, it may be reasonable to hypothesize that the observed difference in the estimated values of the *c* parameter between the EXERT and RELAX conditions reflects a process of precision weighting of the connections among the midbrain, dorsomedial striatum, prefrontal cortex, anterior cingulate, and insular cortex, primarily deployed through the neuromodulatory action of catecholamines. Habit-driven, context-independent behavior has been linked to the activity of the dorsolateral striatum – the posterior putamen in humans (Balleine and O’Doherty, 2010) – within a circuit including sensorimotor and premotor cortices. On the other hand, goal-directed, context-dependent behavior has been associated with the dorsomedial striatum – mainly the caudate nucleus – which is connected to lateral, medial, and orbital prefrontal regions, cingulate cortex, and other associative areas (see, e.g., Buabang et al., 2025; Malvaez, 2020; Tricomi et al., 2009). In Khachouf et al. (2017), the intentional engagement of effort in a Stroop task was associated with a markedly increased activation of the dorsomedial striatum along with other regions mentioned above but, notably, no significant change of activation was observed in the dorsolateral striatum. Although this aligns with the present findings of an increase of the motivation-related parameter *c* – rather than the habit-related parameter *e* – driven by intentional effort, the mapping of the observed effect onto specific neural circuits remains speculative at this stage and will have to be verified by future imaging studies with targeted functional connectivity analyses.

Several factors motivated the decision to model our data using an active inference approach. First, this framework is particularly well suited for studies with limited sample sizes, especially in clinical populations where recruitment may be challenging. This is because it explicitly captures uncertainty in parameter estimates, helping to quantify whether additional data are needed, but also because it uses the full sequence of behavioral measurements for each participant, rather than having to rely on summaries like overall accuracy and average response times. Furthermore, each individual can be well characterized in terms of precise individual estimates, improving the inferences at the group level if between-subject variability is not excessive. Second, active inference allows for the estimation of model parameters representing causal factors that are not directly observable but are meaningfully interpretable, thus making it possible to explicitly test specific hypotheses about behavior and decision-making that depend on such hidden factors. Third, the active inference framework naturally accounts for two sources of variability in behavior: the normal trial-to-trial variation (the choices are sampled from a probability distribution) and a more general random variability implemented via the inverse temperature parameter $\lambda$; this latter feature enables the model to capture phenomena such as distraction, which can lead participants to make errors that do not depend on motivation or task demands. Finally, our operational definition of effort in terms of the divergence of context-dependent actions from context-independent habitual policies – effectively a cost functional – is consistent with current characterizations of effort as a cost-benefit decision process within a neuroeconomic theoretical framework (Kool and Botvinick, 2014; Kurzban et al., 2013; Shenhav et al., 2017).

In summary, we have demonstrated the feasibility of a modeling approach based on active inference to the estimation of parameters related to effort and motivation from behavioral data collected during a Stroop task. While, at least to our knowledge, this is one of the few studies to date in which active inference with model inversion has been applied to recover parameter estimates from actual observed data – and in particular, with the use of a deep temporal model – it is important to recognize some limitations. First, due to the use of a two-level generative model and the collection of hundreds of trials per participant, the computational time required for model inversion is substantial, even when utilizing a high-performance computing system. Second, the model was tested only on healthy participants, thus its applicability to patient populations – an extension we consider both promising and relevant – will need to be separately assessed and may require modifications to the model to accurately reproduce behavior. Third, in our study, the psychological meaning of the *c* parameter could not be directly confirmed by specific first-person ratings targeting motivation (which we did not collect), nor was motivation independently manipulated in the experimental paradigm (e.g., via different amounts of monetary rewards), thus our interpretation will need to be verified by future studies. Fourth, the employed experimental setup did not include a condition with a ‘natural’ (i.e., uninstructed) level of applied effort, thus both the EXERT and the RELAX conditions may represent a deviation from the natural level of effort investment. Fifth, since we did not include text stimuli without semantic content (e.g., the string ‘XXXX’ in colored fonts), we were not able to assess whether the manipulation of intentional effort influenced response facilitation (better performance on congruent trials), interference (worse performance on incongruent trials), or both.

It is also important to note that, while we found evidence for an association between the voluntary deployment of cognitive effort, our model was applied only to a specific cognitive task. Although the Stroop paradigm has often been used to study cognitive effort, the latter can involve various control mechanisms in different tasks or contexts – e.g., suppression of prepotent responses, increased attentional allocation to targets, enhanced suppression of distractors, etc. (Ritz et al., 2022) – thus our findings may not be directly generalizable to all these different scenarios. In thinking about whether, and to what extent, the findings here generalize to other settings and tasks, it is interesting to think about the things for which we might hold preferences. For instance, consider if we had asked participants to specifically exert themselves to perform the task quickly. Here, we might expect to have to define preferences—and habits—over the timings of their responses. While such questions are vitally important in effort research, where speed–accuracy trade-offs are key measures of the deployment of effort, this appears to be a different sort of instruction. In principle, one could model this paradigm and might see a decrease in accuracy as the preference for faster responses increases (or as habitual biases favoring slower, more deliberative responses are suppressed). Furthermore, the choice of the model and its parameters was informed by the general scheme of the active inference framework, leading to the working definition of mental effort as the divergence between habitual actions and context-dependent policies. This is a rather abstract definition and does not get into the details (and, consequently, nor does the model) of how the high-level constructs of motivation and habit exert a causal influence on specific control mechanisms. Indeed, there is evidence for a complex role of cognitive control in the Stroop task through a variety of potential mechanisms (Bugg et al., 2008; Gonthier et al., 2016; Henik et al., 2018), but investigating this was beyond the scope of the present study. To some extent, our model is also agnostic about the mechanisms of deployment of voluntary effort. One could, in principle, propose a further higher level for the model at which decisions about modifications of either automatic response suppression or preference enhancement are made. In other words, these results do not preclude the narrative that “I have a preference for deploying more exertion because I have been asked to, and in doing so will enhance my preferences to suppress the automatic response”. While this is an interesting direction for future research, it would likely be impractical to efficiently fit models of this size to data to answer these questions.

Despite the limitations mentioned above, we think that this work opens up significant opportunities for future research. As mentioned above, it can be potentially extended to clinical populations to investigate how conditions such as fatigue or psychological burnout are related to motivation and effortful engagement, which may have both important diagnostic and therapeutic implications. Furthermore, the computational framework developed in this study could be adapted to other neuropsychological tasks – e.g., the emotional Stroop (Martyr et al., 2011; Tondelli et al., 2022) – facilitating the development of normative models to support neurologists in their clinical practice.

## Supplementary Information

Below is the link to the electronic supplementary material.

Supplementary file 1 (pdf 1179 KB)

## Data Availability

Data are publicly available (https://github.com/riccardo-maramotti/effort-stroop).

## Footnotes

1 In the main text and figures, bold symbols denote vectors and unbolded symbols denote scalars. Overall, our notation conforms to the one used in the active inference book of Parr et al. (2022).

2 The $\sigma$ symbol denotes the softmax function (normalized exponential), which is used to make an arbitrary vector a vector representing a probability distribution (i.e., summing to 1).

3 A one-hot vector is a vector that is zero everywhere except for a single position where it values one. Our response vector has five levels, corresponding to the four different button presses plus a null level, representing the absence of a response. Thus, a response issued by pressing the second button in the button box will be coded as $o_t=[0, 1, 0, 0, 0]$.

4 For instance, the second column assigns 1 to both cells corresponding to the first participant and $-2/(40-2) \approx -0.0526$ to all other cells.

## Code availability

MATLAB code is publicly available (https://github.com/riccardo-maramotti/effort-stroop). In addition, the model on which this analysis is based is already available freely as part of the Statistical Parametric Mapping (SPM25) software package and can be accessed from https://github.com/spm/spm/blob/main/toolbox/DEM/DEMO_MDP_Stroop.m.
