# Supplementary material for: Understanding mechanisms of voluntary engagement of mental effort using active inference
Source: Cogn Affect Behav Neurosci. 2026 Mar 16;26(2):688–702. doi: 10.3758/s13415-026-01419-z (PMC13095915; doi:10.3758/s13415-026-01419-z)
Supplement: Supplementary file 1 — (pdf 1179 KB) [file 13415_2026_1419_MOESM1_ESM.pdf]

Understanding mechanisms of voluntary engagement of mental effort using active inference

Riccardo Maramotti*†, Thomas Parr†, Manuela Tondelli, Daniela Ballotta, Sanjay G. Manohar, Giovanna Zamboni, Giuseppe Pagnoni

*Corresponding author. E-mail: riccardo.maramotti@unimore.it
†These authors contributed equally to this work.

Supplementary Materials

1 Technical details on the active inference model

The model implemented in this study builds on a recently proposed active inference framework for the Stroop task (Parr, Holmes, Friston, & Pezzulo, 2023), with important adaptations tailored to our experimental design. Here, we present the core structure of the model, emphasizing the modifications introduced with respect to Parr et al. (2023). For readers seeking a more comprehensive overview of the original modeling approach, we recommend consulting the cited work. However, Supplementary Figures 1 and 2 are designed to provide some mechanistic intuition which should be sufficient to understand the core principles of the modeling used for this paper.

The model is a hierarchical Partially Observable Markov Decision Process (POMDP), with a higher (*slow*) level that encodes task instructions and sequences, and a lower (*fast*) level that controls trial-specific actions. The active inference scheme takes as input a set of parameters and gives as output a probability distribution over possible actions. From this distribution, we derive predictions about both responses and response times. Supplementary Figure 1 provides a graphical representation of the model's structure: the outcomes of the slow level provide the priors for the states of the fast level. The outcomes of the fast level are the actual observables.

In our POMDP model, the potential actions of participants in response to task instructions are represented by alternative *policies* $\boldsymbol{\pi}$. These policies can be viewed as probabilistic beliefs about the type of response to issue, which subsequently determine the button pressed on the button box. In our case, there are just two available policies, i.e., "report the word text" or "report the font color". Habitual actions — word reading, in this case — are represented by assigning a higher prior probability, which translates to being 'easier' to perform. In contrast, non-habitual actions — like

font-color naming, here — are encoded with a lower prior probability, which corresponds to the requirement of a greater cognitive effort. The policy beliefs are given by the distribution:

$$\boldsymbol{\pi} = \sigma(-\mathbf{E} - \mathbf{G}) \tag{1}$$

where $\mathbf{E}$ and $\mathbf{G}$ are vectors whose elements are the negative log probabilities of each policy, and σ is the softmax function[1]. More specifically, the vector $\mathbf{G}$ represents the expected free energy and each one of its elements can be expressed[2] as $G_\pi = \mathbf{o}_\pi \cdot (\ln \mathbf{o}_\pi + \mathbf{C})$, where the probability distribution $\mathbf{o}_\pi$ describes the expected observations under the policy π, and $\mathbf{C}$ is a negative log-probability vector encoding prior preferences over observations (i.e., large elements of $\mathbf{C}$ indicate that the corresponding observation is aversive relative to those observations with correspondingly smaller elements of $\mathbf{C}$). For the purpose of this model, expected free energy can be regarded as a cost function that reports the risk (i.e., expected deviation from preferences) associated with a given course of action. This means we are effectively working with a form of (constrained) KL-control (Todorov, 2009). In other words, $\mathbf{G}$ scores the implausibility (i.e., improbability given prior beliefs) of each policy, penalizing *risky* courses of action (i.e., whose observations differ from prior preferences, $\mathbf{C}$).

On the other hand, the vector $\mathbf{E}$ quantifies the strength of habitual bias or mental habit. Larger values of $\mathbf{E}$ indicate less habitual policies relative to smaller values. Note that, while the elements of $\mathbf{C}$ score observations, the elements of $\mathbf{E}$ score policies. Cognitive effort can be defined as the divergence between *context-insensitive* prior beliefs ($\mathbf{E}$) and *context-sensitive* beliefs about how to act in the present contingencies ($\mathbf{G}$). Readers seeking further mathematical details on this definition and other equations for POMDPs may want to consult Parr et al., 2023 and Nehrer et al., 2025.

Supplementary Figure 2 illustrates the dynamics of information processing in the model. The policy variable at the highest level in the figure represents decisions about the modality to which we should attend, and not the words and colors themselves. This influences the trajectories of the slow states to form weights for the two modalities (i.e., reading the word or reding the color). These weights influence the fast states so that the distribution of predicted response (from which the measured response is assumed to be sampled) is a weighted combination of the color and word displayed. As this is a probabilistic weighting, the weights assigned to each modality sum to one, meaning facilitation of one modality necessarily means the suppression of the other. In effect, the (potentially opposing) influence of prior habits and preferences jointly determines the relative weights.

Our analysis focused primarily on the probability vectors $\mathbf{C}$ and $\mathbf{E}$ which, as described above, reflect the motivation to perform the task well and the habitual bias toward reading the word (*vs.* stating the font color), respectively. In the computational model, these vectors were expressed via their log-scaled forms, c and e, so that:

$$\begin{aligned} \mathbf{C} &= \exp(c) \cdot [1 \quad -1]^T \\ \mathbf{E} &= \exp(e) \cdot [0.3 \quad -0.3]^T \end{aligned} \tag{2}$$

Higher values of c indicate a stronger preference for accurate performance, while higher values of e indicate a greater strength of the habit to automatically read the word (and thus the need for increased cognitive effort to suppress this habitual response). The interaction between these parameters reflects various individual scenarios, such as cases where a strong motivation for accuracy (c) can mitigate the impact of a strong habitual tendency (e) toward word reading over font color naming.

1.1 Prior parameter distribution for model inversion

Bayesian inference requires the definition of prior distributions for the model parameters. In addition to c and e, our model included two other subject-specific parameters: the inverse temperature λ, dealing with the stochasticity of responses, and a variable α associated with the fastest possible response time for each participant (see the main text for details). All prior distributions were chosen as Gaussian, resulting in normal posteriors. More specifically:

- The prior mean of c was set at 0. In this way, the prior preference (encoded as a probability) for being correct was set at $\exp(2) \approx 7.4$ times the prior preference for being incorrect. Indeed, the prior preference for choosing the 'correct' response modality (the one prescribed by the instructions) at the slow level was defined as $\mathbf{C} = \exp(c) \cdot [1; -1]^T$. As a consequence, a prior value of $c = 0$ corresponds to a log-probability vector $\mathbf{C} = [1; -1]$, where the first event is $\exp(1)/\exp(-1) = \exp(2)$ more probable than the second.
- The prior mean of e was set at 0, and our specification of the $\mathbf{E}$ vector implied that 65% of the time the participant expected to read the word, while 35% of the time she expected to name the color. Indeed, cognitive demand is represented as a log-probability vector $\mathbf{E} = \exp(e) \cdot [0.3; -0.3]^T$. A prior value of $e = 0$ corresponds to a log-probability vector $\mathbf{E} = [0.3; -0.3]$. When applying the softmax operator, this results in a probability of $\exp(0.3)/(\exp(0.3) + \exp(-0.3)) \approx 0.65$ for the first event. Note that in Parr et al. (2023), this vector was defined as $\mathbf{E} = [0.8; -0.8]$, which corresponded to a prior probability of responding to the word text (instead of to the font color) of 85%. In our empirical behavioral data, however, the difference in reaction times between incongruent and congruent trials was smaller than that in the simulations of Parr et al. (2023). Accordingly, we adopted a weaker prior, reflecting a reduced prior bias (65%) toward responding to the word text. In any case, since the prior variance prior was chosen to be relatively large (see below), the end results are driven more by the observed data than by the prior specification.
- In order to ensure the positivity of the parameter representing the stochasticity (or inverse temperature) in the model, we defined $\lambda = \exp(\zeta)$ and modeled ζ using a Gaussian prior with a mean of $\ln(\frac{1}{4})$.
- The parameter α (associated with the lower bound of response times) was modeled using a Gaussian prior of mean $\ln(\frac{1}{2})$.

As for the prior variance of these Gaussian priors, we used $\frac{1}{4}$ for all parameters. This is a rather large value when dealing with log parameters, meaning that we are

not using strong priors. In other words, we are allowing these parameters significant flexibility to deviate from their initial values, ensuring that our model fitting is predominantly guided by the data.

1.2 Posterior predictive check and recovery analysis

To assess whether the active inference model accurately reproduced the observed behavioral patterns, we performed a posterior predictive check. For each participant and each of the eight experimental conditions (*effort* × *target* × *congruence*), we generated simulated behavioral data by sampling from the generative model using parameter estimates obtained from the original model inversion. Simulated data were processed identically to empirical data to compute average response times (RTs) per participant–condition combination. To quantify the agreement between simulated and observed data, we regressed the mean observed RTs on the mean simulated RTs using a Bayesian linear mixed-effects model with random slopes for both participants and experimental conditions. The model yielded a posterior median slope of 0.95 (95% CI [0.74, 1.17]) and an intercept of 0.11 (95% CI [–0.04, 0.26]), indicating a close correspondence without systematic bias. Supplementary Figure 3 reports a scatterplot comparing observed and simulated RTs.

Then, a parameter recovery analysis was performed. For each participant, we simulated behavioral data using the fitted parameters (c and e) and reinverted the model to obtain new parameter estimates. Recovered values were strongly correlated with the original ones ($r = 0.90$, $p < 0.001$ for c; $r = 0.89$, $p < 0.001$ for e), indicating excellent preservation of the inter-individual ranking. A Bland–Altman analysis further revealed negligible mean bias (–0.08, 95% limits of agreement [–0.81, 0.48] for c; –0.01, 95% limits of agreement [–0.70, 0.68] for e), suggesting no systematic over- or underestimation across the parameter space.

Note that also in Parr et al., 2023 the authors simulated data based on various combinations of prior mean values for c and e. Then, they inverted the generative model and the estimates were compared to the hypothesized values of c and e. Their results showed that the recovered estimates for the c parameter were in good agreement with the original ones; for the e parameter, while recovery was relatively poor, the rank order was preserved (i.e. higher hypothesized values of e were associated with higher posterior values of e). Also, it is worthwhile to note that the values chosen in Parr et al., 2023 for the prior mean of the parameters were relatively small, ranging between -0.25 and 0.25. In contrast, the estimated values for the parameter in this study moved away from zero. This might be due to a number of factors (including our specific manipulation of engaged effort), and it justifies the application of a new recovery analysis on our real behavioral data.

2 Within-subject analysis on the Stroop effect

A three-way repeated-measures ANOVA was performed to investigate the effect of *effort* (EXERT vs. RELAX), *target* (color-naming vs. word-naming), and *congruency* (congruent vs. incongruent), as well as their interactions, on median response times. The results are shown in Supplementary Table 2.

The analysis revealed a significant main effect of *effort*, indicating that participants responded faster during EXERT compared to RELAX trials ($p < 0.001$). A robust main effect of *congruency* was also observed, with slower responses for incongruent relative to congruent stimuli ($p < 0.001$). No significant main effect of *target* was found ($p = 0.559$). Importantly, a significant interaction between *effort* and *congruency* ($p = 0.017$) suggests that the Stroop effect was modulated by effort, with a reduced interference effect in the EXERT condition. The interaction between *target* and *congruency* did not reach significance, although the result approached the threshold ($p = 0.063$), indicating a trend toward differential interference between color-naming and word-naming tasks.

To further investigate these effects, contrast analyses were performed comparing the Stroop effect (incongruent vs. congruent) across the four combinations of *target* and *effort*. Also, the effect of effort on the Stroop effect was tested. Results are reported in Supplementary Table 1. The Stroop effect was found to be present for both levels of *target* and both levels of *effort*. However, the effect of effort on the Stroop effect was significant only in the color-naming task. Thus, only in the color-naming task the intentional increase in applied cognitive effort reduced the interference typically observed in the Stroop effect.

3 Correlation between model parameters and NASA-TLX ratings

To examine whether the active inference parameters captured participants' subjective experience, we correlated the estimates of c and e with NASA-TLX sub-scales collected after each run. Analyses were performed separately for the RELAX and EXERT condition, and also on the difference between RELAX and EXERT. Results are shown in Supplementary Tables 3 and 4. We did not observe any significant relationship. This was not too surprising, however, as correlations between first-person reports and third-person measurements are very rarely revealed Dang, King, and Inzlicht, 2020. Furthermore, we should also consider that the subjective ratings were collected after each run, and thus reflected an overall assessment over both the word-reading and the color-naming tasks. This makes it difficult to interpret the correlations between the model parameters and the subjective ratings because, e.g., a weaker habit for reading the text may be facilitating in the color-naming task (and thus lead to subjective reports of less effort and frustration), but have an opposite effect in the word-reading task (and thus lead to subjective reports of greater effort and frustration).

References

Dang, J., King, K. M., & Inzlicht, M. (2020). Why are self-report and behavioral measures weakly correlated? *Trends in Cognitive Sciences*, *24*(4), 267–269. doi:10.1016/j.tics.2020.01.007

Nehrer, S. W., Ehrenreich Laursen, J., Heins, C., Friston, K., Mathys, C., & Thestrup Waade, P. (2025). Introducing activeinference.jl: A julia library for simulation and parameter estimation with active inference models. *Entropy*, *27*(1). doi:10.3390/e27010062

Parr, T., Holmes, E., Friston, K. J., & Pezzulo, G. (2023). Cognitive effort and active inference. *Neuropsychologia*, *184*, 108562. doi:https://doi.org/10.1016/j.neuropsychologia.2023.108562

Todorov, E. (2009). Efficient computation of optimal actions. *Proc Natl Acad Sci U S A*, *106*(28), 11478–11483. doi:10.1073/pnas.0710743106

Footnotes

1. The σ symbol denotes the softmax function (normalized exponential), which is used to make an arbitrary vector a vector representing a probability distribution (i.e., summing to 1).

2. In general, there is an additional term in the expected free energy dealing with the expected entropy of the conditional distribution representing the beliefs about how hidden states generate observations, but this is suppressed here as it is a constant for our model.

Supplementary tables and figures

Supplementary Table 1: Contrast analysis examining the Stroop effect (incongruent vs. congruent response times) across *effort* and *target* conditions.

Contrast	Estimate	df	t	p
color: EXERT (incong. vs. cong.)	0.103	19	8.625	< 0.001
color: RELAX (incong. vs. cong.)	0.146	19	6.269	< 0.001
word: EXERT (incong. vs. cong.)	0.071	19	6.713	< 0.001
word: RELAX (incong. vs. cong.)	0.083	19	3.549	0.002
color: RELAX (incong. vs. cong.) - EXERT (incong. vs. cong.)	0.044	19	2.306	0.033
word: RELAX (incong. vs. cong.) - EXERT (incong. vs. cong.)	0.012	19	0.732	0.473

Supplementary Table 2: Within-subject effects obtained from a three-way repeated measures ANOVA.

Within-subject effect	Estimate	df	F	p
Effort	0.360	19	21.880	< 0.001
Target	0.005	19	0.353	0.559
Congruency	0.406	19	89.065	< 0.001
Effort * Target	0.001	19	0.310	0.584
Effort * Congruency	0.008	19	6.870	0.017
Target * Congruency	0.022	19	3.896	0.063
Effort * Target * Congruency	0.002	19	1.200	0.287

Supplementary Table 3: Pearson correlations and p-values between NASA-TLX sub-scales and parameter c in RELAX, EXERT, and RELAX-EXERT conditions.

Sub-scale	RELAX	EXERT	RELAX-EXERT
Mental demand	−0.14 (p = .542)	−0.06 (p = .817)	0.36 (p = .115)
Physical demand	0.06 (p = .803)	0.11 (p = .644)	−0.12 (p = .630)
Temporal demand	0.27 (p = .256)	0.05 (p = .838)	0.18 (p = .446)
Effort	−0.05 (p = .848)	−0.14 (p = .564)	0.06 (p = .790)
Performance	−0.33 (p = .154)	−0.20 (p = .391)	0.08 (p = .724)
Frustration	0.24 (p = .301)	0.17 (p = .465)	0.11 (p = .652)

Supplementary Table 4: Pearson correlations and *p*-values between NASA-TLX sub-scales and parameter *e* in RELAX, EXERT, and RELAX-EXERT conditions.

Sub-scale	RELAX	EXERT	RELAX-EXERT
Mental demand	0.16 ($p = .502$)	0.02 ($p = .935$)	0.22 ($p = .349$)
Physical demand	0.01 ($p = .975$)	−0.23 ($p = .321$)	−0.41 ($p = .072$)
Temporal demand	0.20 ($p = .396$)	−0.17 ($p = .478$)	−0.23 ($p = .323$)
Effort	0.12 ($p = .603$)	0.20 ($p = .398$)	−0.14 ($p = .563$)
Performance	−0.37 ($p = .108$)	−0.15 ($p = .530$)	0.30 ($p = .200$)
Frustration	−0.20 ($p = .397$)	−0.28 ($p = .235$)	−0.27 ($p = .252$)

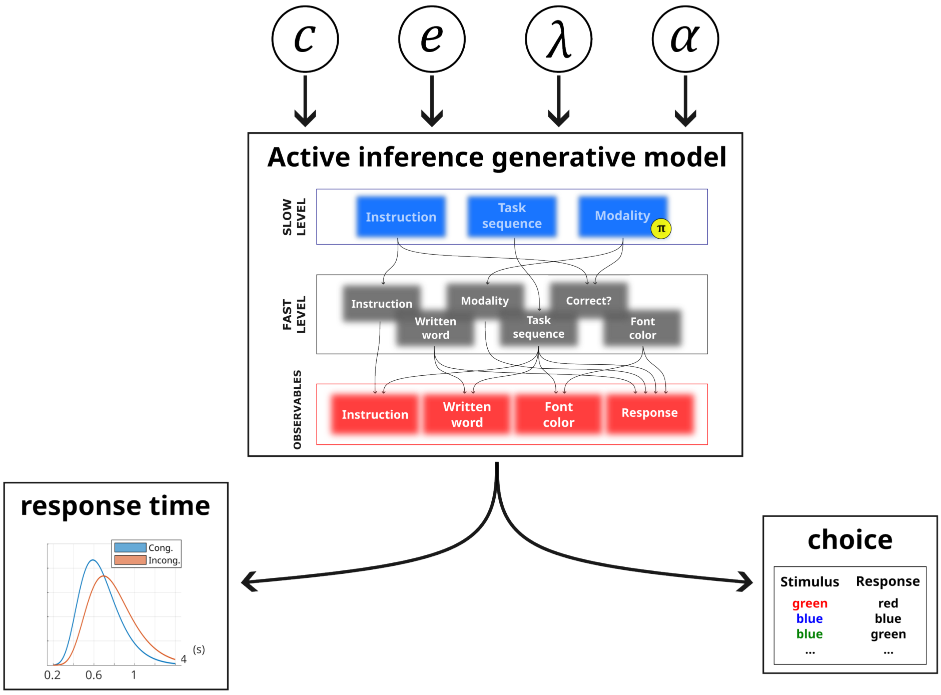

Supplementary Fig. 1: Hierarchical structure of the generative model, with a higher (slow) level encoding the task instructions and sequence, and a lower (fast) level encoding trial-specific features. The outcomes of the slow level function as priors for the fast level and, in turn, the outcomes of the fast level correspond to the observable data. Arrows indicate statistical dependencies: only the boxes related by a statistical dependency are linked by an arrow. At the slow level, the model features three hidden states factors: an *instruction* state, which determines whether the task involves reading words or identifying font color; a *task sequence* state, which changes from an instructional to a response context; a *response modality*, which is the only policy-dependent term in the generative model and determines the strategy without influencing the observables directly. Actions that affect directly the observables are specified at the fast level, while the outcomes of the factors of the slow level correspond to three states of the fast level with the same labels. There are three other state factors in the fast level: the *color* of the font; the *written* word; and a state that reports the *correctness* of the chosen mental action, comparing the instruction and response modality at the slow level. Crucially, this state has no influence over the generated observations. Given the values of the parameters c, e, λ and α (see main text), the generative model provides a probabilistic estimation of the action $\mathbf{u}(c, e, \lambda, \alpha)$ — i.e., a vector function whose elements are the probabilities for each possible action conditioned upon the parameters — enabling the simulation of both choice and response time data. Indeed, the graph in the bottom-left represents the probability distributions of response times according to the computed confidence in the next response choice, while the inset in the right middle row shows a simulated response choice for the 'report the font color' condition.

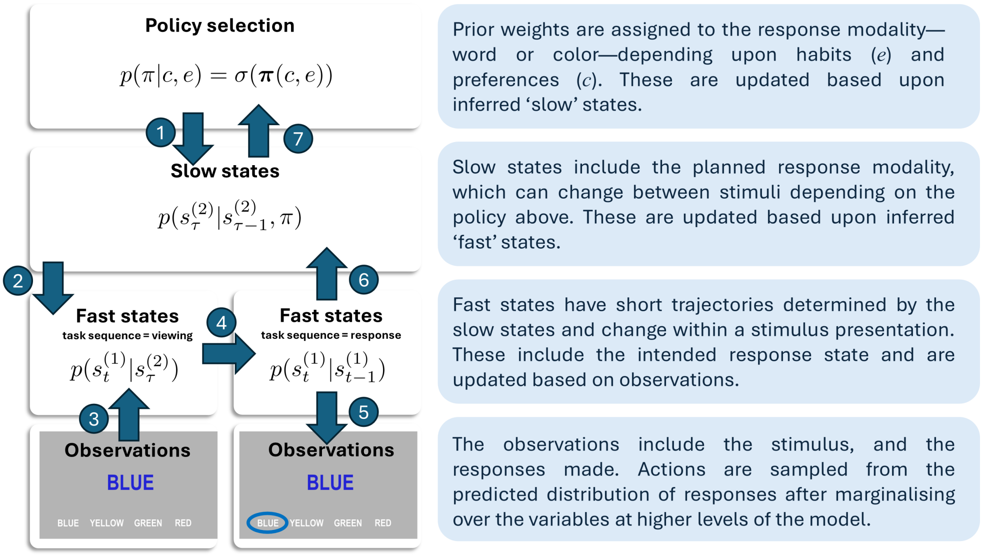

Supplementary Fig. 2: This figure is designed to provide some mechanistic detail on how the model assumes participants solve the task of deciding how to respond to a given stimulus presentation for the Stroop task. This is designed to be an accompaniment to Supplementary Figure 1, which details more specifics of how this model was set up to estimate behavior in our analysis. Each level is annotated with a brief description. From the perspective of someone performing the task, we assume she has prior weightings as to which modality she expects to respond with, which are used to contextualize (1) transitions in intended response modalities from stimulus to stimulus as represented in slow level states. These states provide prior weighting (2) for faster states that deal with the transition from a new stimulus to a behavioral response (e.g., observing the blue stimulus to declaring it blue). The prior (2) and observational evidence (3) are combined through variational inference to update these faster states. Still in the fast level, after transitioning (4) from a viewing to a response state, the response is sampled (5) from a predictive distribution (which depends upon 1-4) over possible responses (in this example, 'blue' is the selected response). The fast states inferred at this stage can be used to update the slower states (6) and the policy (7) through variational inference such that revised priors can be brought to bear on the next stimulus presentation.

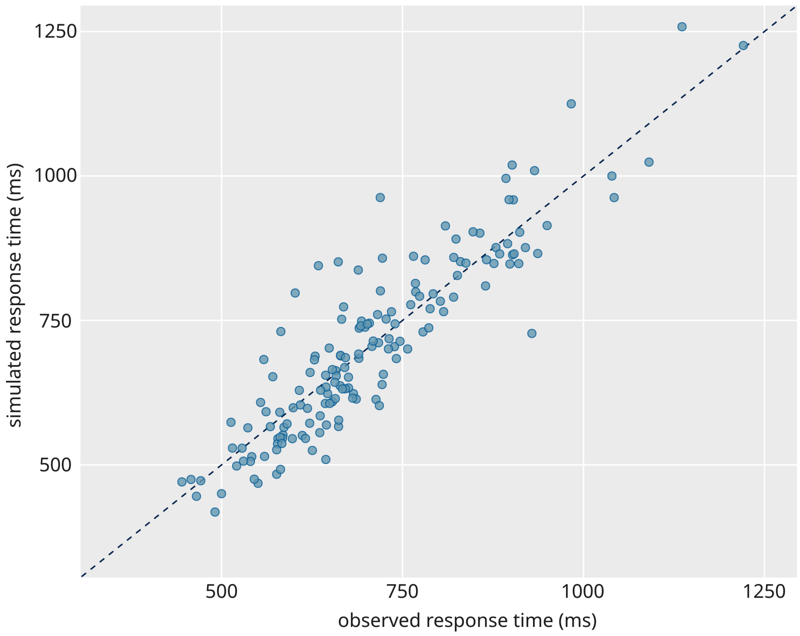

Supplementary Fig. 3: Scatterplot comparing mean observed RTs and corresponding simulated RTs across all participants and experimental conditions (each point represents one participant–condition combination). The dashed diagonal line indicates perfect agreement between observed and simulated data.
